# Clinical study on post evaluation after listing of Qizhi Weitong granules: Study protocol clinical trial (SPIRIT compliant)

**DOI:** 10.1097/MD.0000000000019758

**Published:** 2020-04-17

**Authors:** Lin Xu, Jiaqi Zhang, Juanjuan Li, Lin Lv, Zedan Zhang, Fengyun Wang, Xudong Tang

**Affiliations:** a China Academy of Chinese Medical Sciences; b Department of Gastroenterology, Xiyuan Hospital of China Academy of Chinese Medical Sciences; c Beijing University of Chinese Medicine, Beijing, China.

**Keywords:** functional dyspepsia, protocol, Qizhi Weitong granule, randomized controlled trial, traditional Chinese medicine

## Abstract

**Background:**

Functional dyspepsia (FD) is a highly prevalent functional gastrointestinal disorder which brings a significant impact on patients’ quality of life. Although there are many available treatments to alleviate dyspepsia symptoms, most of them are far from satisfactory. Traditional Chinese medicine (TCM) has shown good potential in the treatment of FD, especially in terms of improving symptoms and adverse effects of Western medicine. Qizhi Weitong granule (QZWTG), a TCM preparation, has been utilized in treating FD for a long time and has achieved good clinical results. However, the existing evidence of its efficacy and mechanism of action is insufficient. Hence, the purpose of this study is to evaluate the efficacy and safety of QZWTG in the treatment of FD.

**Methods:**

This study is a multicenter, randomized, double-blinded, double-placebo, positive drug parallel controlled clinical study. The experiment will be carried out in 8 hospitals at the same time, and a total of 384 cases of participants will be randomly assigned to the experimental group and the control group (n = 192). The experimental group will be given QZWTG and Mosapride citrate tablet placebo, and the control group will be given QZWTG placebo and Mosapride citrate tablet. After 4 weeks of intervention and 2 weeks of follow-up, the efficacy and safety of QZWTG in patients with FD will be observed. The primary outcomes are the change in the main symptom score. The secondary outcomes include TCM syndrome evaluation, the change of the Hamilton anxiety scale and the Hamilton depression scale, and advanced events. This study will explore the biological mechanism of QZWTG in the treatment of FD through the results of blood and urine metabolomics.

**Discussion:**

This trial will provide first-hand evidence on whether QZWTG is noninferior to Mosapride citrate tablet. There will be a new option for the treatment of FD if noninferiority is set up. In addition, the efficacy and safety of QZWTG in the treatment of FD will be evaluated, and the mechanism of QZWTG in the treatment of FD will be explored through the metabolomics of blood and urine. On the other hand, as far as we know, this study may be the largest trial of efficacy and safety of QZWTG in the treatment of FD, which has important application value.

## 1 Introduction

Functional dyspepsia (FD) is one of the most common functional gastrointestinal disorders,^[1]^ which is characterized by a series of dyspepsia symptoms such as postprandial fullness, early satiation, epigastric pain, in the absence of structural explanation.^[2]^ The overall global prevalence of dyspepsia has been estimated to be 20% to 40%.^[3]^ In Rome IV, according to the difference of main symptoms, FD can be divided into 2 subgroups: postprandial distress syndrome (PDS) and epigastric pain syndrome (EPS), also, the 2 subgroups can overlap.^[2]^ A recent study shows that approximately 10% of the adult population from the USA, Canada, and the UK fulfills symptom-based criteria for Rome IV criteria of FD.^[4]^ Although FD is not life-threatening, it brings a significant impact on patients’ quality of life.^[5]^

At present, FD is considered to be caused by multiple factors, which may be related to abnormalities of gastric motility, gastric hypersensitivity, brain-gut interaction,^[6]^ duodenal acid,^[7]^ duodenal bile, duodenal barrier defect, and immune activation.^[8]^ Gastric motility disorder and sensorimotor abnormality are recognized as one of the main factors leading to FD, and prokinetics are recommended for the treatment of FD.^[2,9]^ Although, a number of randomized controlled clinical studies have shown that the efficacy of prokinetic agents,^[10–12]^ such as Cisapride, in patients with FD is significantly higher than that of placebo, which can significantly alleviate the symptoms of dyspepsia,^[13]^ such as upper abdominal fullness,^[14]^ but the clinical application of it is limited due to the side effects of heart.^[15]^ In addition, existed treatment options may be effective for FD, but their therapeutic effect is far from satisfactory. Due to the high prevalence of FD and the lack of effective treatments, FD is now a considerable clinical problem for the healthcare system.^[16]^

Traditional Chinese medicine (TCM) has shown good potential in the prevention and treatment of FD, especially in terms of relieving symptoms and adverse effects of Western medicine.^[17,18]^ Therefore, it is necessary to seek a supplementary therapeutic approach for the treatment of FD.

QZWTG is a TCM preparation and is commercially developed to relieve epigastric distress and pain. QZWTG is composed of 6 kinds of Chinese herbals (Table 1): Radix Bupleuri (Chaihu), Rhizoma Corydalis (Yanhusuo (baked)), Fructus Aurantii (Zhike), Nutgrass Galingale Rhizome (Xiangfu (baked)), Radix Paeoniae Alba (Baishao), and Radix Glycyrrhizae Preparata (Zhigancao). All herbs were tested to the same standard. QZWTG is developed by the China Resources Sanjiu Pharmaceutical Co., Ltd: No.1 Guanqing Road, Guanlan Hightech Park, Guanhu Street, Longhua District, Shenzhen, Guangdong, China and is approved for production on the market. The drug approval No.: gyzz21021522. At present, QZWTG has been approved by the State Food and Drug Administration for clinical treatment in Gastrointestinal motility and sensory disorders related diseases,^[19,20]^ and have achieved good results. QZWTG has the functions of soothing the liver, regulating qi, relieving pain in the stomach, and used to relieve symptoms such as epigastric dull pain, fullness, acid regurgitation, nausea, vomiting, reduction of appetite, and noise in heart and mouth. Previous research^[21]^ has shown that QZWTG has a significant symptomatic improvement over placebo in patients with PDS. Moreover, QZWTG has been proved that it has the effect of improving gastric motility,^[22–26]^ and has no side effects such as the central nervous system and cardiovascular system.^[21]^ However, the consistency of this effect remains uncertain. In addition, there is a lack of comparative studies between QZWTG and existing treatment methods, such as Mosapride, a prokinetic agent.

**Table 1:**
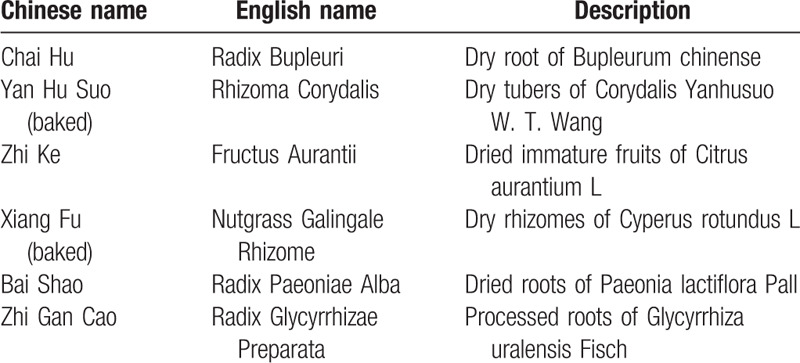
Main components of Qizhi Weitong granules.

The aim of this randomized, double-blinded, double-placebo, positive drug parallel controlled, multicenter clinical trial was to further verify the therapeutic effect of QZWTG compared with Mosapride in patients with FD, and observe the effectiveness and safety of QZWTG on different subtypes of FD, so as to guide clinical practice.

## 2 Methods

### 2.1 Study design

This trial is a multicenter, randomized, double-blinded, double-placebo, positive drug parallel controlled clinical trial. A total of 384 patients will be enrolled and randomly assigned to 1 of 2 groups: Qizhi Weitong and Mosapride group. After 1 week of the screening period, 4 weeks of intervention, and 2 weeks of follow-up, the effectiveness, and safety of QZWTG will be evaluated by comparing the various indicators of the 2 groups, including clinical symptoms, biochemical indicators. This study will be conducted with the principles of the Declaration of Helsinki and Good Clinical Practice guidelines, and the Consolidated Standards of Reporting Trials 2017 for Chinese herbal medicine (CHM) recommendations.^[27]^

**Figure 1:**
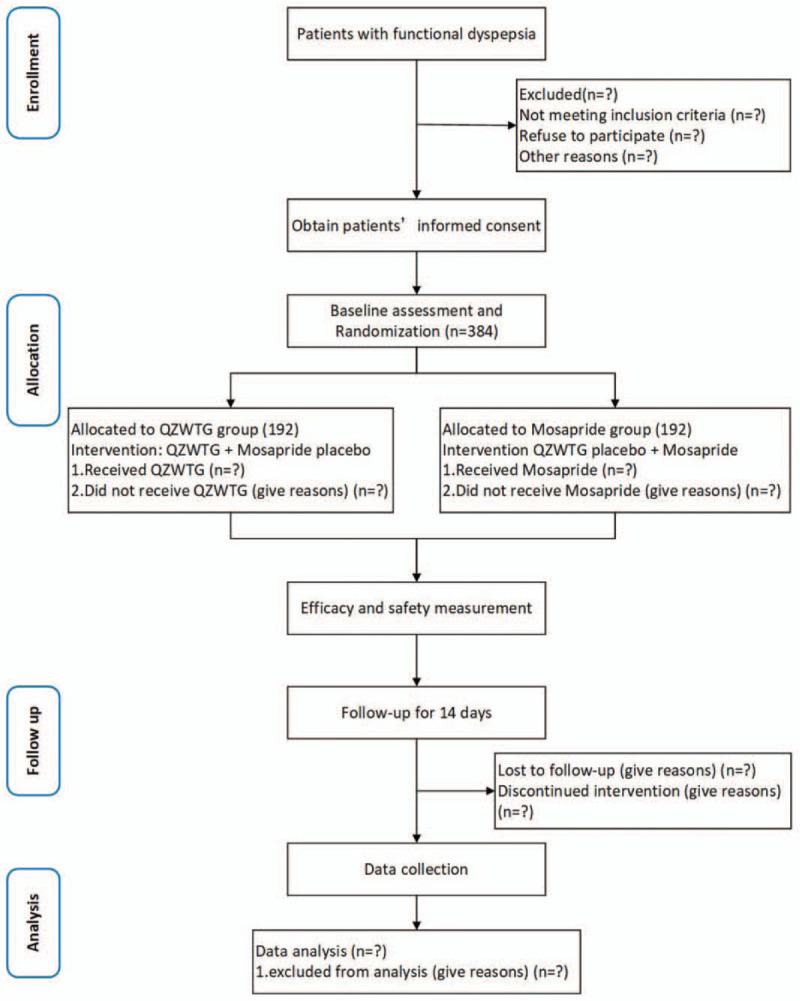
CONSORT flow diagram for QZWTG. CONSORT = clinical practice guidelines, and the consolidated standards of reporting trials, QZWTG = Qizhi Weitong granule.

As the leading unit of the research, Xiyuan hospital is responsible for training the standard operating procedures (SOPs) of researchers and supervising the progress of all clinical sites. Other participating units include the Beijing Friendship Hospital Affiliated to Capital Medical University, the First Affiliated Hospital of Guangzhou University of Traditional Chinese Medicine, the First Affiliated Hospital of Zhejiang University, Shengjing Hospital Affiliated to China Medical University, the First Affiliated Hospital of Henan University of Traditional Chinese Medicine, Jiangsu People's Hospital, Zhongshan Hospital affiliated to Fudan University. Recruitment allocation: 384 cases will be competition among centers. The Consolidated Standards of Reporting Trials flow diagram of this trial is briefly illustrated in Figure 1. This study protocol is registered with the ClinicalTrials (NCT03149393).

### 2.2 Participant recruitment

Inpatients and outpatients with FD from the 8 participating hospitals will be enrolled. Two experienced gastroenterologists will screen the patients according to the inclusion and exclusion separately. Patients assessed by 2 doctors meeting the criteria of the trial will be included in the trial. In addition, the recruitment advertisements for this trial will be in the form of posters and webpages in the 8 research units for public recruitment. It mainly includes a brief introduction of the drugs, treatment methods, and the rights and interests of the patients in this study. For those who refuse to participate, we will record their basic information and the reasons for refusing to participate. The trial started in January 2017 and will end in December 2020.

### 2.3 Diagnostic criteria

#### 2.3.1 Diagnostic criteria of FD

The diagnostic of FD will refer to the criteria published in Rome IV^[2]^:

(1) One or more of the following: A. The discomfort of fullness after a meal; B. early satiety; C. upper abdominal pain; D. upper abdominal burning.
(2) There is no evidence of structural disease that can explain the above symptoms, and no organic disease is found by gastroscopy or barium contrast examination of the upper gastrointestinal tract, and the examination time is after the occurrence of symptoms;
(3) The symptoms appeared at least 6 months before diagnosis, nearly 3 months met the above diagnostic criteria.

#### 2.3.2 Inclusion criteria

Patients must meet both of the inclusion criteria of the screening period and treatment period.

Screening period criteria:

(1) Meeting the diagnostic criteria of FD in Rome IV;
(2) Age 18 to 65 years old, gender is not limited;
(3) The subjects were informed, and the subjects voluntarily signed informed consent;
(4) The subjects have the reading ability.

Treatment period criteria:

(1) Patients continued to be willing to participate in the study;
(2) After treatment in the screening period, the sum of at least 1 symptom score (degree + frequency) is still ≥4 points, and the reduction rate is ≤50%.

#### 2.3.3 Exclusion criteria

Patients who meet 1 or more of the exclusion criteria listed below will not be allowed:

(1) Patients suffering from gastric ulcer, gastroscopy see bleeding and mucosal erosion, pathological examination showed atrophy of gastric mucosa, intestinal metaplasia or dysplasia;
(2) Patients with *Helicobacter pylori* infection positive;
(3) Patients with gastroesophageal reflux disease.
(4) Patients with digestive system organic lesions.
(5) The patient had a history of stomach or abdominal surgery.
(6) Patients had taken the relevant drugs in the past 2 weeks.
(7) Patients suffering from a severe illness affecting survival.
(8) Pregnant or lactating women.
(9) Participating in clinical trials of other drugs.
(10) Long term using of sedative-hypnotics.
(11) Suspected or true alcohol, drug abuse history.

### 2.4 Removal, dropout, and termination criteria

#### 2.4.1 Withdrawal of participants

Participants can voluntarily drop out at any time during the trial. All the participants who have filled in the informed consent form and screened for qualified entry into the trial, no matter when and why they quit, as long as they have not completed the prescribed observation period, are all cases of dropout. When participants drop out, the researcher should ask the reason, record the last time of taking medicine, complete the evaluation items that can be completed, and fill in the case report form (CRF) fall off reason table.

#### 2.4.2 The withdrawal decided by the investigator

(1) Participants with serious adverse events.
(2) During the experiment, the participants had some complications or special physiological changes, so they were not suitable to continue the experiment.
(3) In the trial, the participants used the banned drugs specified in the trial protocol due to poor compliance.
(4) According to the judgment of the doctor, there might be dangerous events. To protect the participants, they should be removed from the clinical trial and receive other treatments, and the case will be treated as invalid.

All selected cases, no matter whether they fall off or not, should record and keep the research medical record and CRF. All the report forms of the lost cases are submitted to the clinical responsible unit for summary and statistical analysis.

#### 2.4.3 The criteria for the termination of clinical trials

(1) Failure of random masking;
(2) The rate of unblinding was more than 20% of the sample size;
(3) All follow-up evaluations were completed.

### 2.5 Sample size calculation

According to previous study, the effective rate of Mosapride in the treatment of FD was around 60%.^[28–30]^ According to the ratio of test group: control group = 1:1, class I error *α* was 0.025 (1 side), class II error *β* was 0.2 (80%), and noninferior standard Δ was set as 13% to estimate the sample size, 160 participants are required for each group. Considering the possibility of missing data and loss of follow-up (20% in total), we will recruit a total of 384 participants for the 2 groups, with 192 participants in the experiment group and 192 participants in the control group.

### 2.6 Randomization

In this study, the dynamic random method was used, each center competed for the group, and the clinical trial electronic central random system (DAS for IWRS 5.0, provided by Beijing Bozhiyin Technology Co., Ltd.: Room 2-2209, Times Fengfan Building, 15 Majiapu West Road, Fengtai District, Beijing, China) was used to assign the random number. The influencing factors were the subtype of FD, such as EPS, PDS, EPS + PDS. After the subjects pass the screening, the random applicants (investigator/drug administrator/CRC) log in Das for interactive web response system (IWRS) to apply for the random number and distribute the drug according to the drug package number displayed in Das for IWRS.

### 2.7 Blinding

In order to ensure the quality of clinical research, double-blind design was used in this clinical study. Since QZWTG and Mosapride are different drug formulations respectively, a double simulation was used to make it difficult for participants to find out which drug is a placebo. To avoid the subjective bias of researchers and subjects and minimize the known bias sources, the data analysis of the whole experiment adopts the third-party blind evaluation. Electronic emergency letters were used in this test. In case of emergency, the researchers think that knowing the drugs taken by the subjects is conducive to the treatment of adverse events, the blind can be opened urgently through Das for IWRS. Researchers and participants in this trial will be asked to fill out a questionnaire about what treatments were taken at the last visit to assess the success rate of blinding.

### 2.8 Interventions

Volunteers who meet the inclusion criteria will be required to provide written consent to participate in the trial. They will then undergo a physical screening test to determine if there are other comorbidities that may impact the trial. After the successful screening, they will be allocated to receive QZWTG or Mosapride citrate tablets according to a random sequence. A baseline measurement for each participant is then performed, including clinical symptoms scores and TCM syndrome scores. The study flow chart is shown in Figure 1. All outcome measurements will be taken by medical workers who are familiar with the management of the assessments and will be unaware of the participants’ group assignments.

The trial group will be given QZWTG (2.5 G each time) and Mosapride citrate tablet placebo (5 mg each time). The control group will be given QZWTG placebo (2.5 G each time) and Mosapride citrate tablet (5 mg each time). All of them will be given 3 times a day, 30 minutes before breakfast, lunch, and dinner. The QZWTG placebo consists of starch without any active ingredient is produced by the same manufacturer as QZWTG. It is a dextrin that matches as much as possible the appearance and taste of QZWTG. The drug instructions for QZWTG and placebo are completely consistent. Both the drug and the corresponding placebo had the same outer packaging, color, shape, and taste so that neither the participants nor the researchers could identify which intervention the participants were receiving. The packaging of the drug will be returned to the investigator at the end of the treatment.

### 2.9 Comorbidities and exacerbations regulation

Participants are allowed to remain their originally basic treatment (such as medicine for hypertension) taken before recruited. If there is an unexpected situation, we will deal with it immediately. In addition, other Chinese or Western medicines for FD should be prohibited.

### 2.10 Data collection

#### 2.10.1 Background information

Background information includes demographic data and general clinical data, which will be recorded before the start of the 1-week screening period. Demographic data include age, gender, height, weight, occupation, and so on. General clinical data include a course of the disease, treatment history, combined disease, and medication, etc. The information and privacy of the participants will be strictly protected from the public.

#### 2.10.2 Safety outcomes

The safety was evaluated by routine physical examination and laboratory examination, which will be checked before and after the intervention, to access the safety of QZWTG on patients with FD. Routine physical examination includes body temperature, respiration, blood pressure, and heart rate. Laboratory tests include blood routine, urine routine, and stool routine, stool occult blood, liver function, renal function, abdominal B ultrasound, gastroscopy, and electrocardiogram (ECG). Safety evaluation will be performed before and after the intervention. Any adverse event occurs during the study will be observed and recorded in detail.

#### 2.10.3 Primary and secondary outcomes

The primary outcomes are the change of the main symptom score contains an evaluation of postprandial fullness discomfort, early satiety, epigastric pain, epigastric burning sensation and so on. The main symptom score mainly utilized to assess the severity of symptoms and distinguish between EPS and PDS. The score will be assessed at baseline and week 2, week 4, week 6.

Owing to psychosocial factors likely play a role in FD,^[31–33]^ with greater FD symptom severity correlated with depression and somatization, this trial also includes the anxiety and depression assessment form in the assessment of secondary outcomes. The secondary outcomes mainly include TCM syndrome evaluation, the change of the Hamilton anxiety scale and the Hamilton depression scale, which will be measured at baseline and every 2 weeks during the treatment period to evaluate the changes of patients’ mental and psychological status, biochemical indicators, and changes of blood metabonomics and urine metabonomics (Table 2).

**Table 2:**
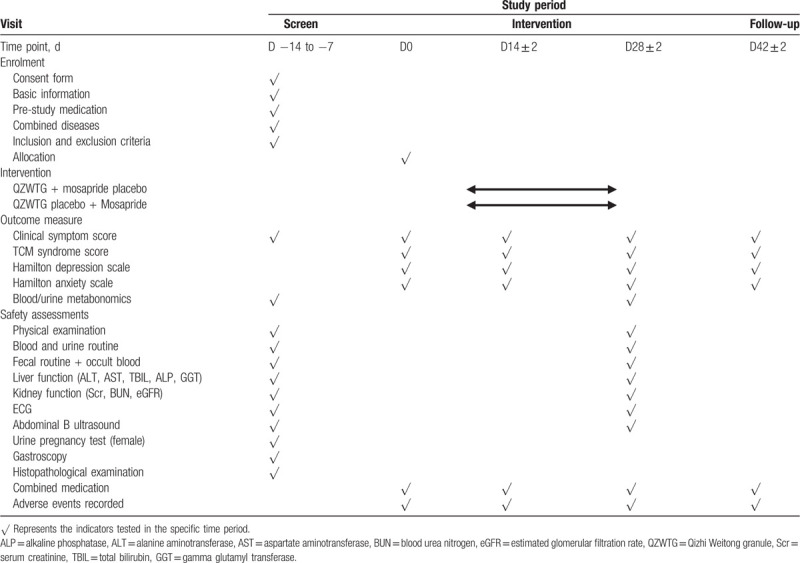
Data recording nodes from baseline to follow-up visits.

### 2.11 Quality control and data management

Before the study, clinicians, statistical and methodological experts were invited to review and revise the study protocol many times. To ensure the accuracy and integrity of clinical data, the researchers should be trained on the consistency of scale evaluation to ensure that relevant personnel fully understand the research scheme and SOPs. The participants were selected in strict accordance with the plan, and the communication with the participants was strengthened to ensure the follow-up and medication compliance. All data is first recorded on paper CRF by researchers, and then double input electronic data acquisition system by electronic means. The whole process will be supervised by an independent quality inspector.

### 2.12 Statistical analysis

Analysis data set including full analysis set (FAS), per-protocol set and safety analysis set.

(1) FAS refers to the case that has used the research drug at least once and has at least 1 main index measurement data after treatment. For the case data of failure to observe the whole treatment process, the last observation data is used to carry forward to the final result last observation carried forward (LOCF) of the study.
(2) Per-protocol set: good compliance (treatment compliance ≥80%), no drug prohibition during the study, complete all follow-up, no serious violation of the study protocol.
(3) Safety analysis set including all cases randomly enrolled and at least once used the study drug.

A detailed description of the cases of exfoliation and rejection, including the time and cause of exfoliation or rejection. Descriptive statistics indicate mean, standard deviation, maximum, minimum, median, confidence interval, frequency (constituent ratio), and so on. Per-Protocol (PP) analysis and FAS analysis were carried out for the main efficacy indexes at the same time; CMH method was used for the multicenter counting data, and *t* test and variance analysis were used for the measurement data; for the confounding factors that were difficult to control or not controlled before treatment, such as the imbalance between groups before treatment, least squares mean (LSMEAN) and 95% confidence limit or Logistic regression of analysis of covariance (ANCOVA) were used as covariates to determine the difference of curative effect among groups was eliminated. In addition to the main efficacy indicators, the superiority test was carried out with a statistical superiority test (Δ = 0). In the comprehensive analysis of TCM symptoms, the specific clinical manifestations (post-meal swelling, early satiety, epigastric pain, epigastric burning) and other symptoms were analyzed respectively. The statistical test was bilateral difference test, and those with *P* less than or equal to .05 were considered statistically significant. Statistical analysis will be performed by the SAS 9.2 software.

### 2.13 Patient and public involvement

Neither patients nor their family members were involved in the study design. The results of the studies will be published in public to benefit policy-makers, clinicians, and patients.

### 2.14 Ethics and dissemination

This trail has been approved by the Ethics Committee of Xiyuan Hospital, China Academy of Chinese Medical Sciences, and this trial is will be reviewed annually. This trial has been registered at ClinicalTrials.gov on May 11, 2017, NCT03149393. The results of this study will be disseminated to the public through academic conferences and peer-reviewed journals.

## 3 Discussion

Function dyspepsia is a common, functional disease, influencing millions worldwide.^[34]^ It seriously impairs patients’ social activities, daily activities, and quality of life.^[35]^ The high economic burden of FD to families and societies is obvious.^[36,37]^ The extensive clinical experience of using Chinese medicine in the treatment of FD in China shows that TCM preparations are effective. Due to the complexity of the pathogenesis of FD,^[1]^ the recent use of TCM has attracted more and more attention, providing new prospects for TCM, which be considered effective in improving clinical symptoms. TCM possesses the advantages of being simple, convenient, efficient, inexpensive, and without serious adverse reactions.

QZWTG is a TCM preparation commercially developed to relieve epigastric distress and pain. Pharmacological studies have shown that QZWTG has the effect of curing atropine-induced gastrointestinal motility dysfunction mice.^[22]^ However, whether QZWTG is effective in patients with FD still needs further confirmation. Therefore, we design this critical clinical trial, which has the following advantages:

(1) This study is designed as a randomized, double-blind, placebo-controlled, clinical, critical trial from the perspective of evidence-based medicine, that is, considered to be the most definitive research method of treatment evaluation;
(2) At the same time, this is a multicenter trial be carried out in 8 comprehensive third-grade first-class hospitals across China, which improves the external validity and representativeness of the sample and reduces the risk of selection bias;
(3) We use double-blind and double placebo method to better reflect the authenticity of the experiment.
(4) Because clinical symptoms are often accompanied by a certain degree of subjectivity, we combine the core symptoms (upper abdominal pain, burning sensation of upper abdomen, discomfort of fullness, and early satiety after meal) score and TCM syndrome score to ensure scientific objectivity;
(5) To ensure quality, all staff in the study must complete the training of the SOPs of the research before recruitment;
(6) As the main phase of the trial will be performed at home, practitioners will maintain good communication with the participants by phone after discharge.

However, the design of the trial also has potential limitations, for example, the 4-week treatment period and the 2-week follow-up period are a bit short. In addition, our trial will be carried out in 8 regions of China, it is uncertain whether the research results are applicable to other populations.

In conclusion, the aim of this study is to answer whether QZWTG can be used to treat FD, relieve clinical symptoms of FD, and provide objective data about effectiveness and safety. If the trial is successful, it will provide patients and physicians with a new option for better disease remission, which can be implemented on a larger scale in clinical and community settings. As an innovative and potentially cost-effective strategy, this approach can reduce the financial burden of FD. Given the high prevalence of FD, the results of this study can be used to provide information on future international guidelines.

## 4 Trial status

Recruitment started in March 2017 and is expected to finish in December 2020, 46 months in total.

## Acknowledgments

The authors would like to acknowledge China Resources Sanjiu Pharmaceutical Co., Ltd for produce and package of granules. Most especially, the authors would like to thank the patients with functional dyspepsia who participate in this study.

## Author contributions

**Conceptualization:** Lin Xu, Fengyun Wang, Xudong Tang.

**Data curation:** Jiaqi Zhang, Juanjuan Li, Zedan Zhang.

**Funding acquisition:** Fengyun Wang, Xudong Tang.

**Methodology:** Lin Xu, Jiaqi Zhang.

**Project administration:** Juanjuan Li, Lin Lv.

**Resources:** Lin Xu, Fengyun Wang.

**Supervision:** Jiaqi Zhang, Lin Lv.

**Writing – original draft:** Lin Xu.

**Writing – review & editing:** Lin Xu.

Lin Xu orcid: 0000-0003-2487-6528.
